# Stunning fish with CO_2_ or electricity: contradictory results on behavioural and physiological stress responses

**DOI:** 10.1017/S1751731115000750

**Published:** 2015-05-11

**Authors:** A. Gräns, L. Niklasson, E. Sandblom, K. Sundell, B. Algers, C. Berg, T. Lundh, M. Axelsson, H. Sundh, A. Kiessling

**Affiliations:** 1 Department of Animal Environment and Health, Swedish University of Agricultural Sciences, Box 234, 53223 Skara, Sweden; 2 Department of Animal Nutrition and Management, Swedish University of Agricultural Sciences, Box 7024, 75007 Uppsala, Sweden; 3 Department of Biological and Environmental Sciences, University of Gothenburg, Box 463, 40530 Gothenburg, Sweden

**Keywords:** welfare, teleost, aquaculture, narcosis, slaughter

## Abstract

Studies that address fish welfare before slaughter have concluded that many of the traditional systems used to stun fish including CO_2_ narcosis are unacceptable as they cause avoidable stress before death. One system recommended as a better alternative is electrical stunning, however, the welfare aspects of this method are not yet fully understood. To assess welfare in aquaculture both behavioural and physiological measurements have been used, but few studies have examined the relationship between these variables. In an on-site study aversive behaviours and several physiological stress indicators, including plasma levels of cortisol and ions as well as blood physiological variables, were compared in Arctic char (*Salvelinus alpinus*) stunned with CO_2_ or electricity. Exposure to water saturated with CO_2_ triggered aversive struggling and escape responses for several minutes before immobilization, whereas in fish exposed to an electric current immobilization was close to instant. On average, it took 5 min for the fish to recover from electrical stunning, whereas fish stunned with CO_2_ did not recover. Despite this, the electrically stunned fish had more than double the plasma levels of cortisol compared with fish stunned with CO_2_. This result is surprising considering that the behavioural reactions were much more pronounced following CO_2_ exposure. These contradictory results are discussed with regard to animal welfare and stress physiological responses. The present results emphasise the importance of using an integrative and interdisciplinary approach and to include both behavioural and physiological stress indicators in order to make accurate welfare assessments of fish in aquaculture.

## Implications

Many methods used to stun and kill fish in aquaculture result in poor animal welfare. One of the strategic actions in EUs ‘Strategy for animal welfare’ is therefore to evaluate fish welfare in aquaculture (European Commission, 2012). The present study compared several indicators of stress in fish stunned using CO_2_ or electric field exposure before slaughter. The results showed that contradictory conclusions on the impact on fish welfare can be reached depending on what physiological and/or behavioural measurements are included in the assessment. An integrative and interdisciplinary approach is therefore called for when assessing fish welfare in aquaculture.

## Introduction

According to the food and agricultural organization of the United Nations, aquaculture represents the fastest-growing animal-based food production sector in the world (FAO, 2013). Over 44 million tons of finfish are produced annually, which includes >200 fish species and is estimated to include between 37 and 120 billion individuals (FAO, 2013, http://fishcount.org.uk). This means that the number of fish slaughtered at fish farms every year exceeds that of all other farmed vertebrates combined (http://faostat.fao.org). While the World Organization for Animal Health (OIE) and the European Food and Safety Authority (EFSA) include fish in their directives and recommendations on animal welfare; standards on global or EU level on how to handle, stun and kill fish in aquaculture are currently lacking. Consequently, farmed fish are today often handled and killed using methods that do not comply with welfare recommendations of OIE and EFSA (EFSA, 2004 and 2009a; Lines and Spence, 2012; van de Vis *et al.*, 2012; OIE, 2014). Therefore, one of the relatively few strategic actions in the EU’s Strategy for animal welfare is to further investigate and evaluate fish welfare in aquaculture (European Commission, 2012).

Theoretically, the magnitude of an animal welfare problem can be assessed by multiplying the *severity* of an animal’s suffering by its *duration* and *number* of individuals affected (Broom, 1991; Webster, 2001; van de Vis *et al.*, 2012). However, even this relatively simple method becomes challenging when applied to aquaculture conditions. First, harvest of fish is only reported in tonnes, and so all reported numbers of individuals are highly speculative (http://fishcount.org.uk). Another problem is the rather limited knowledge about how fish perceive and react to pain. Although it is known that fish have nociceptors (Sneddon *et al.*, 2003), their ability to experience the sensations of pain and suffering is still intensely debated making it problematic to estimate the *severity* of a welfare issue (Rose *et al.*, 2012). In terrestrial farm animals a range of behavioural and physiological indicators associated with stress, pain and suffering are often used to quantifying the *severity* of a welfare hazard (Broom, 1991; Webster, 2001). Fortunately, many of the commonly used physiological stress indicators (e.g. stress hormones and cardiovascular stress responses) are conserved among fish and other animals (Wendelaar Bonga, 1997; Barton, 2002), but the behavioural repertoire of fishes is much less studied compared with mammals and it is difficult to translate behavioural stress indicators between species (Martins *et al.*, 2012). Consequently, information available in the peer reviewed literature regarding fish welfare in relation to slaughter is rather limited (EFSA, 2004 and 2009b; van de Vis *et al.*, 2012). To fill these knowledge gaps it is important to apply an integrative and interdisciplinary approach. Yet, few studies have examined the relationship between physiological and behavioural welfare indicators in farmed fish (EFSA, 2009c). Although many stunning systems have been ruled out as ethically unacceptable (see Table 1), our present knowledge on how aquaculture species react to the suggested alternatives remain limited.

**Table 1:** The main systems of stunning and killing fish and a summary of the recommendations provided by the World Organization for Animal Health and the European Food and Safety Authority

System	Recommendation
CO_2_ narcosis	
Asphyxia in air or on ice	
Live chilling	
Salt bath	Should not be used to kill fish because they cause avoidable suffering before death
Ammonia solution	
Electro-immobilization^1^	
Decapitation	
Evisceration of live fish	
Mechanical stunning^2^	Can be appropriate for some species
Electrical stunning	

1 Includes physical exhaustion using electrical shocks.

2 Includes percussive stunning, spiking and shooting.

One common system for stunning fish in aquaculture is still narcosis by immersion in carbon dioxide (CO_2_) saturated water (Robb *et al.*, 2000; EFSA, 2009a). This method has been heavily questioned from an animal welfare point of view, mainly because it is known that exposure to CO_2_ triggers aversive behaviours in fish (Marx *et al.*, 1997; Robb and Kestin, 2002; van de Vis *et al.*, 2003). Other serious problems associated with CO_2_ narcosis is that it may leave the animal immobilized before consciousness is lost (Robb *et al.*, 2000), and that it elicits a strong primary stress response with release of cortisol and catecholamines (Bernier and Randall, 1998; Sandblom *et al.*, 2013; Seth *et al.*, 2013).

The use of electric field exposure has been highlighted as a promising alternative to stun fish in aquaculture (van de Vis *et al.*, 2003; EFSA, 2009a; Lines and Spence, 2012; OIE, 2014). Electric stunning can be achieved both in water (wet stunning) and out of water (dry stunning). In fact, as the critique against many of the traditional stunning systems (e.g. CO_2_) has intensified, many fish farms have adopted electrical stunning systems instead (Lines *et al.*, 2003; Lines and Spence, 2012). Despite the recent popularity of this method the welfare aspects of electrical stunning are still not well understood. Indeed, if the electrical stunning is insufficient it may leave the animal electro-immobilized, which means that it is paralyzed but not unconscious, and therefore still subjected to pain and stress (Kestin *et al.*, 1995 and 2002; Robb *et al.*, 2000; Robb and Kestin, 2002).

Few previous studies have examined the relationship between behavioural and physiological stress responses to assess welfare in aquaculture. Therefore, we choose to compare both behavioural and physiological stress indicators in Arctic char (*Salvelinus alpinus*) stunned with a traditional CO_2_ system and a modern electrical dry stunning system. The study was conducted at a fish farm where the two systems where running in parallel in an attempt to emulate a true situation. The overall aim of the study was to assess if dry electrical stunning is better than stunning with CO_2_ from a welfare perspective. Specifically, we examined escape behaviours and recovery time following stunning to assess the severity of the stressor and the duration of the stunning. In addition, stress physiological variables including plasma cortisol, haematological variables (haematocrit (Hct), haemoglobin concentration (Hb) and mean corpuscular haemoglobin concentration (MCHC)), plasma electrolytes (Na^+^, Ca^2+^ and K^+^) and osmolality were examined.

## Material and methods

### Animals

The experimental part of the study was conducted in November 2013 at Umlax AB’s (www.umlax.se) facilities for Arctic char (*S. alpinus*) at Slussfors, located along river Umeälven in Swedish Lapland. The study was conducted on fish with a mass range of 478 to 1286 g (mean: 768±23 g) and a fork length of 32.4 to 44.3 cm (mean: 38.7±0.3 cm). Fish were held in net pens (Ø12 m) in the river and during the 1-week period of the study the mean river temperature was 4.6±0.2°C. All experimental protocols were approved by the Ethical Committee of Gothenburg (permit 177–2013).

### Description handling and stunning routines at Slussfors

Approximately 1 week before slaughter, feeding ceased and the net pen was transported to the shore. On the day of slaughter, the bottom of the net pen was elevated in order to increase the density of fish. The fish were then transferred by a large dip net into a tank containing water that had been bubbled with CO_2_ until near saturation (>1 h). After the fish had lost equilibrium and were deemed unconscious (~10 min), a metal grid lifted the fish on to an open surface. Personnel then manually cut the throat of individual fish and transferred them to an adjacent water tank for exsanguination. The processing of all fish in one net pen typically takes 1 to 2 weeks. Remaining fish are therefore repeatedly exposed to crowding as new groups of fish are netted from the pen.

### Experimental protocols

**Figure 1:**
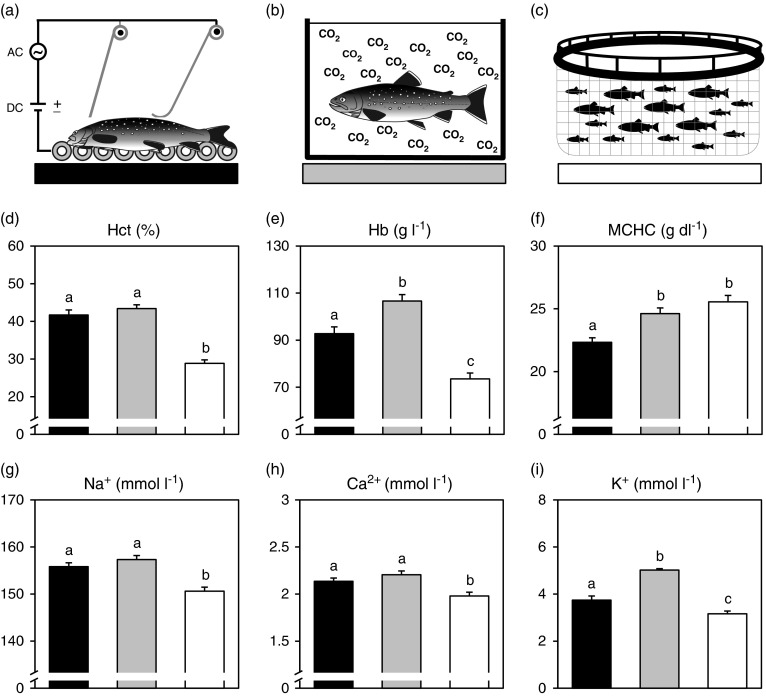
Haematological variables and plasma ion in Arctic char (*Salvelinus alpinus*) stunned with dry electric exposure (a, black), CO_2_ exposure in water (b, grey), or controls immediately netted from the net pen (c, white). Data for haematocrit (d), haemoglobin concentration (e), mean corpuscular haemoglobin concentration (f), sodium ion concentration (g), calcium ion concentration (h) and potassium ion concentration (i) are presented as means and error bars show s.e.m. Different letters indicate significant differences among treatments (*P*<0.05).

A commercial dry electrical stunning system from Seaside AS (www.stansas.no) was mounted in parallel with the traditional CO_2_ system and controlled by a trained operator. The electrical system exposed the fish to a combination of ~80 V direct current and ~10 V alternating current at a frequency of 100 Hz for ~10 s while the fish is transported along an automatic conveyor belt (Figure 1a). The settings were chosen in accordance with manufacturers recommendations for stunning of Arctic char.

The study was designed as a two part experiment. In the first part, aversive behaviours and recovery times after stunning with the two systems were evaluated (*n*=10 per treatment). To standardize the experimental protocols, and minimize bias from routines before stunning, fish in the two stunning experiments were treated identically. Experimental fish were caught manually with dip nets from the net pens (10 fish each in two trials) and transferred into a 100 l transportation tank (within seconds to minimize air exposure). The tank was then transported the distance of ~25 m between the pier and the slaughter house where the fish were netted over to the respective stunning system (again within seconds to minimize air exposure). The time until loss of equilibrium was determined as the time elapsed from the netting from the transport tank until equilibrium was lost in the two stunning systems. The recovery time was defined as the time it took for the stunned fish to regain equilibrium after being moved from the stunning exposure into a tank filled with aerated water (Bernier and Randall, 1998).

The second part of the experiment was designed to assess the physiological stress responses in fish (*n*=20 per treatment) exposed to a 10-min long period of CO_2_ stunning to mimic normal routines at the farm or a 10 s electrical stunning using the alternative Seaside system. These responses were compared with a control group that was netted immediately from the net pen (Figure 1a to c). After the three treatments all fish were euthanized by a sharp cranial blow and blood was sampled from the caudal vessels using heparinized syringes. The blood sampling occurred immediately after the respective stunning procedure, or immediately after netting from the pen in the control group. Capture and transportation of fish was performed identically to the procedures in the first part of the experiment and care was taken to expose all fish to comparable netting times across experimental treatments. For the electrical stunning, fish in each of the two trials were divided into two groups of five fish to ensure sufficient time for blood sampling.

### Analyses of blood and plasma

Hct (%) was determined by centrifugation of whole blood using heparinized micro capillary tubes and Hb (g/l) was determined using a haemoglobin analyser (HemoCue 201þ, Ängelholm, Sweden), calibrated for fish blood (Clark *et al.*, 2008). MCHC (g/dl) was calculated as Hb/Hct×10. Remaining blood was centrifuged and the plasma collected, frozen on dry ice and stored at −80°C for further analysis. Plasma cortisol (ng/ml) was analysed using a radioimmunoassay described by Young (1986) validated by Sundh *et al.* (2011) and using a cortisol antibody (Code: S020; Lot: 1014–180182) purchased from Guildhay Ltd (Guildford, Surrey, UK). As tracer, hydrocortisone-[1,2,6,7–3H(N)] (NET 396; NEN Life Sciences Products, Boston, Massachusetts, USA) was used and cortisol standards were prepared from hydrocortisone (Sigma, St. Louis, MO, USA). The determination of the radioactivity was performed with a Wallac 1409 liquid scintillation counter. Plasma concentration (mmol/l) of the electrolytes sodium (Na^+^), potassium (K^+^) and calcium (Ca^2+^) were measured using a flame photometer (Eppendorf 003310004; EppendorfeNetheler Hinz GmbH, Hamburg, Germany) with serum standard solution as standard. Samples and standards were diluted in 5 mM LiCl. Plasma osmolality (mOsmol/kg) was determined by the freezing point method using an Advanced Instruments 3MO osmometer (Advanced Industries, Inc., Wichita, Kansas, USA).

### Statistical analysis

Statistical analyses were conducted in SPSS 20 for Windows (SPSS Inc., Chicago, IL, USA). All variables were compared among groups using one-way ANOVA followed by Sidak *post hoc* tests. Plasma cortisol and osmolality values were log transformed before analysis to meet the assumptions for normal distribution and equal variances. In the groups exposed to electrical stunning all variables were tested for time effects by correlating their consecutive number with the dependent variable. All variables where differences were *P*<0.05 are regarded as statistically significant. Data is presented as means±s.e.m.

## Results

No correlations were found between sampling order and any of the measured variables in fish entering the electrical stunning system indicating that sampling order had no effect on the recorded variables.

### Behavioural responses and post-stunning recovery time

CO_2_ stunning caused rapid and violent aversive reactions, including attempts to escape from the tank and uninterrupted circulatory swimming behaviours. It typically took 2 to 4 min for fish to loose equilibrium and none of the fish recovered after the 10 min CO_2_ exposure. In contrast, electrical stunning caused an instantaneous reaction, resulting in the mouth and opercula flared open followed by tetanus-like immobility. All fish recovered equilibrium within 4 to 7 min following the electric exposure.

### Haematological variables

Both Hct and Hb were significantly elevated (*P*<0.0001) following the two stunning treatments compared with the control group (Figure 1d and e). The CO_2_ exposed group also had a significantly (*P*=0.002) higher blood haemoglobin concentration compared with the group exposed to electricity (Figure 1e). MCHC was significantly lower in the group exposed to electricity than the group stunned with CO_2_ (*P*=0.002) and the control group (*P*<0.0001; Figure 1f).

### Plasma electrolytes and osmolality

Plasma levels of Na^+^ (*P*<0.0001), Ca^2+^ (*P*<0.05) and K^+^ (*P*<0.05) were consistently significantly lower in the control group compared with the two treatment groups (Figure 1g to i). The plasma K^+^ level was also significantly lower in the group exposed to electricity compared with the group stunned with CO_2_ (*P*<0.0001, see Figure 1g). The patterns in plasma ion levels were also reflected in the plasma osmolality where both the group exposed to electricity (338.3±2.1 mOsm/l, *P*=0.001) and CO_2_ (342.6±3.4 mOsm/l, *P*<0.0001) had significantly higher osmolality compared with the control group (325.4±1.5 mOsm/l).

### Plasma cortisol

**Figure 2:**
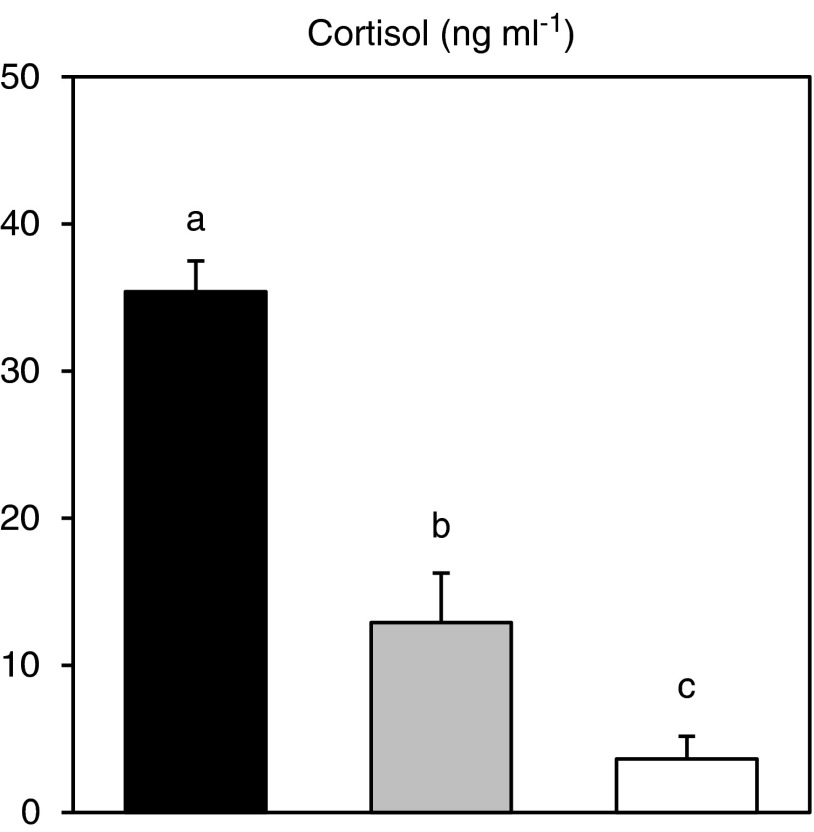
Plasma levels of cortisol in Arctic char (*Salvelinus alpinus*) stunned with dry electric exposure (black), CO_2_ in water (grey) or controls netted immediately from the net pen (white). Data are presented as means and error bars show s.e.m. Different letters indicates significant differences among treatments (*P*<0.05).

Plasma cortisol levels were significantly elevated in the two treatment groups compared with the control group (electricity, *P*<0.0001 and CO_2_, *P*=0.003; Figure 2). However, when comparing the two stunning treatments plasma cortisol levels were significantly higher in the group exposed to electricity compared with the group stunned with CO_2_ (*P*<0.0001; Figure 2).

## Discussion

The present study show contradictory results between behavioural and physiological welfare indicators in Arctic char (*S. alpinus*) stunned with CO_2_ exposure or electricity.

### Initial observations of fish behaviours

Visual observations of the two stunning systems at the fish farm at Slussfors, suggested several potential welfare benefits of using dry electrical stunning instead of the traditional CO_2_ system. In accordance with previous studies conducted on a range of fish species including Arctic char, aversive escape behaviours were seen in all fish exposed to water saturated with CO_2_ (Marx *et al.*, 1997; Robb and Kestin, 2002; Lines *et al.*, 2003; van de Vis *et al.*, 2003; Seth *et al.*, 2013). When stunning with electricity no escape behaviours were observed and instead the immobilization was nearly instantaneous. These results are in agreement with previous behavioural observations in fish stunned with electricity (Robb *et al.*, 2000; Kestin *et al.*, 2002; Robb and Kestin, 2002; Lines *et al.*, 2003; Sandblom *et al.*, 2012). In addition, CO_2_ stunning is known to cause excessive mucous production making the fish slippery and more difficult to handle (Marx *et al.*, 1997). The handling problem is further complicated by the high amounts of water resulting from moving the fish from the CO_2_ saturated water to the surface used for throat cutting. In fact, this handling problem after CO_2_ stunning is likely the main reason why up to 20% of manually performed throat cuts are reported to be unsuccessful (EFSA, 2004 and 2009a; Kiessling *et al.*, 2013). In contrast, with dry electrical stunning, there is no increased mucous production and during transport of the fish along the short automatic conveyor belt excessive water is lost improving the work environment at the area of throat cutting (A.G., personal observation). From the on-site observations it was also noted that the close to instantaneous effect of electrical stunning eliminated the somewhat arbitrary evaluation on whether the fish is unconscious or not, which typically needs to be performed by the personnel during CO_2_ narcosis (Robb *et al.*, 2000; van de Vis *et al.*, 2003; Martins *et al.*, 2012). Collectively, these practical and technical advantages could improve the welfare of farmed fish by minimizing human errors, such as missed throat cuts and false decisions of unconsciousness.

Even so, a potential welfare hazard associated with the electrical stunning was noted during the on-site observation as it appears that the duration of the insensibility following electrical stunning may not always be long enough. For example, in Atlantic salmon, brain death (as defined by the onset of brain dysfunction using Electroencephalography) from bleeding following cutting of the ventral aorta takes up to 7 min at 6°C (Robb *et al.*, 2000). Thus, with the relatively transient effects of the dry electrical stunning observed here, where only 4 to 7 min was required for Arctic char to regain equilibrium, there appears to be a considerable risk that fish may recover consciousness before death occurs from exsanguination.

### Stress physiological responses

Plasma cortisol is the most commonly used primary stress indicator in fish and plasma levels typically rise rapidly a few min after exposure to a stressor and may remain elevated for several hours (Wendelaar Bonga, 1997; Barton, 2002). The cortisol levels in our control group are comparable to levels previously reported for uninstrumented Arctic char (Lyytikainen *et al.*, 2002; Pottinger, 2010), and considerably lower than levels reported for isolated fish instrumented with dorsal catheters (Sandblom *et al.*, 2012; Seth *et al.*, 2013). While significantly elevated relative to the control, the plasma cortisol levels in the two stunned groups where low compared with previous reports from stressed Arctic char (Lyytikainen *et al.*, 2002; Pottinger, 2010, Sandblom *et al.*, 2012 and 2013; Seth *et al.*, 2013). These discrepancies are probably partly explained by the fact that our fish where uninstrumented, but also because samples were collected immediately after stunning as plasma cortisol levels can continue to increase for up to 90 min after an acute stress event (Lyytikainen *et al.*, 2002; Pottinger, 2010; Sandblom *et al.*, 2012; Seth *et al.*, 2013). Plasma cortisol levels obtained from post-slaughter blood samples is a common method used in various farmed animals including fish to assess welfare implications of various stunning systems (including CO_2_ narcosis and electrical stunning) (Lambooy, 1985; Shaw and Tume, 1992; Hambrecht *et al.*, 2004; Linares *et al.*, 2008; Xu *et al.*, 2011; Sandblom *et al.*, 2012). Nonetheless, the more than two-fold higher cortisol level in the group stunned with electricity compared with the group stunned with CO_2_ was surprising, although it is consistent with studies in lambs comparing responses after CO_2_ narcosis and electric stunning (Linares *et al.*, 2008).

The increase in plasma concentrations of electrolytes and osmolality observed after stunning are clear indications of stress in freshwater fish. The rationale behind this is suggested to be an osmotic flow of water from extracellular to intracellular compartments (i.e. haemoconcentration) due to increased intracellular levels of lactate during stress (Wood, 1991; Wang *et al.*, 1994). The only difference in plasma ion levels observed between the two stunning methods examined here was a higher K^+^ concentration in the CO_2_ group. Beyond the possible effects on plasma electrolytes from increased intracellular lactate levels, elevated plasma K^+^ is also considered a sign of haemolysis and/or tissue cell damage. Thus, it seems likely that the violent escape behaviours observed in the CO_2_ exposed group may have induced cell lysis as shown in mammals during intense exercise (Smith, 1995).

The increases in Hct and Hb following stunning are all common responses to acute stress in fish that is mainly caused by a combination of interrenal humoral release of catecholamines (adrenaline and noradrenaline), as well as increased sympathetic nervous activity causing splenic contraction and release of circulating erythrocytes. However, Hct may also increase from red blood cell swelling (as evident from a reduced MCHC), in part caused by *β*-adrenergic stimulation of red blood cells from circulating catecholamines (Nikinmaa and Huestis, 1984; Wendelaar Bonga, 1997). Fish exposed to CO_2_ had both elevated Hb and MCHC compared with the fish from the group stunned with electricity, suggesting that the elevated Hb and Hct in the CO_2_ exposed group was mostly caused by sympathetic neural stimulation of the spleen (Pearson and Stevens, 1991).

In contrast, the lower MCHC of the electrically stunned group also indicates increased levels of circulating catecholamines causing swelling of red blood cells through a *β*-adrenergic mechanism (Nikinmaa, 1983). Even so, high levels of circulating catecholamines after electrical stunning as a primary stress response should probably be interpreted with caution, because it is known from studies in mammals that isolated chromaffin cells release catecholamines in response to direct electrical stimulation (Alamo *et al.*, 1991). How much this potential mechanism would affect the results of the present study are unknown, but if plasma catecholamine levels and the physiological responses they result in are caused by the stunning technique *per se*, irrespective of the stress perceived by the animal, they may not be suitable for welfare assessments.

### Comparison of behavioural and physiological welfare assessments

Our results show that contradictory conclusions on fish welfare can be reached depending on what physiological or behavioural measurements are included in the assessment. As expected, both stunning systems resulted in significantly higher physiological stress responses compared with the control, but somewhat surprisingly the much less pronounced behavioural response during electrical stunning compared with CO_2_ exposure did not translate into a reduced physiological stress response. Instead, indications of higher stress levels were found in several of the physiological variables after electrical stunning. We see two possible explanations for these seemingly contradictory results.

One possible explanation is that the physiological measurements are biased by the stunning methodology. For example, if the stunning technique affects the investigated variable independent of the stress perceived by the animal, results from blood samples collected after the stunning may lead to erroneous conclusions. For example, as mentioned above, electrical stimulation in mammals directly stimulates catecholamine release from chromaffin tissue (Alamo *et al.*, 1991; Shaw and Tume, 1992). While this could explain the red blood cell swelling after electrical stunning in char reported here, it is unlikely to explain the elevated cortisol levels as no similar release mechanism has been reported for that hormone (Shaw and Tume, 1992).

A second possible explanation would be if the fish where not stunned, when exposed to the electrical system, but rather electro-immobilized. Several studies where brain function has been measured through evoked responses on the electroencephalogram have shown that if the electrical stunning is insufficient it may in fact leave the animal electro-immobilized but still conscious (Kestin *et al.*, 1995; Robb *et al.*, 2000; Kestin *et al.*, 2002; Robb and Kestin, 2002). On the other hand, if the field strength is ‘too high’ or the frequency is ‘too low’ it can cause injuries, like fractures of the vertebrae and blood spots (Lines *et al.*, 2003). Consequently, one of the greatest challenges for developers of commercial electrical stunning equipment is to avoid carcass damage while still being able to guarantee that the welfare of the stunned animals is not compromized.

Thus, when assessing welfare it is crucial to differentiate between stunning and electro-immobilization as the welfare of an animal is only impaired when it is conscious (EFSA, 2009c). Whether this explains the more profound physiological stress response in the fish exposed to electricity in the present study is presently unknown. However, this clearly suggests that future studies should include measurements of consciousness as knowledge gaps in this area are currently preventing accurate welfare assessments of fish during stunning.

## Concluding remarks

The present study confirms that CO_2_ narcosis is an inferior method to stun Arctic char, as it induces violent aversive behavioural escape reactions that will negatively affect fish welfare. When using the dry electrical stunning system no escape reactions were seen, although this technique elicited more pronounced physiological stress responses compared with CO_2_ narcosis. These seemingly contradictory results can presently not be fully explained, but it is clear that further studies assessing consciousness is needed to fully evaluate the welfare effects of different stunning techniques.
